# Research on Utilizing Phosphorus Tailing Recycling to Improve Acidic Soil: The Synergistic Effect on Crop Yield, Soil Quality, and Microbial Communities

**DOI:** 10.3390/plants14223475

**Published:** 2025-11-14

**Authors:** Chuanxiong Geng, Huineng Shi, Jinghui Wang, Huimin Zhang, Xinling Ma, Jinghua Yang, Xi Sun, Yupin Li, Yi Zheng, Wei Fan

**Affiliations:** 1 Institute of Agricultural Environment and Resources, Yunnan Academy of Agricultural Sciences, Kunming 650201, China; gengchuanxiong@163.com (C.G.);; 2 College of Resources and Environment, Yunnan Agricultural University, Kunming, 650201, China; 3 Yunnan Open University, Kunming 650500, China

**Keywords:** phosphorus tailings, acid soil, soil improvement, soil quality, crop yield

## Abstract

Phosphate tailings (PTs) are typical industrial byproducts that can rapidly neutralize soil acidity. However, their acid-neutralizing efficacy, long-term application optimization mechanisms, and high-yield regulation pathways for crops remain unclear. This study conducted a corn-potato crop rotation field trial on acidic soils, investigating the effect of different PT application rates (T: CK, 0 t·ha^−1^; PTs-1, 6 t·ha^−1^; PTs-2, 9 t·ha^−1^; PTs-3, 15 t·ha^−1^) in a multiple cropping system (C: late autumn potatoes (LAP)-early spring potatoes (ESP)-summer maize (SM)). The results showed that two consecutive applications of 9 t·ha^−1^ of PTs produced optimal results, increasing the LAP yield by 12.82% and the soil quality by 76.51%, while improving the ESP soil quality by 46.21%. The higher yield was mainly attributed to a significant increase in the soil pH (0.72–1.58 units) and enhanced chemical and biological properties (higher exchangeable calcium (ExCa), exchangeable magnesium (ExMg), the total exchangeable salt base ion (TEB), and catalase (CAT) and urease (UE) content and lower soil exchangeable acidity (EA), exchangeable hydrogen ion (ExH), and exchangeable aluminum (ExAl) levels). Notably, a synchronized increase in the total phosphorus (TP) and total potassium (TK) during LAP cultivation, combined with simultaneous growth of TP, available nitrogen (AN), and available phosphorus (AP) during ESP cultivation, and a significant increase in TP and AP during SM cultivation, effectively promoted crop yield. Furthermore, continuous PT application significantly enriched phosphorus (P)-soluble functional bacteria, such as Actinomycetes and Chloroflexota, and enhanced the stability of bacterial-fungal cross-boundary networks. In summary, optimal acidity levels and favorable soil texture improved soil quality, consequently increasing corn and potato yields. This study reveals for the first time that PTs can substantially increase crop production via a synergistic mechanism involving acid-base balance, structural improvement, and microbial activation. Not only does this provide a novel strategy for rapidly improving acidic soils, but it also establishes a solid theoretical and technical foundation for utilizing PT resources.

## 1. Introduction

Global climate change, acid rain, and soil acidification due to long-term fertilizer application have severely restricted the sustainable development of agriculture. Statistics show that acidic soils account for 40% of the global total arable land and 21% of that in China [1]. These areas continue to expand, indicating that soil acidification has become a key constraint on global agricultural development [2]. The loss of alkaline metal ions, such as calcium (Ca) and magnesium, in soil increases hydrogen ions and activated aluminum ions, which are the primary causes of soil acidification. Soil acidification reduces nitrogen utilization efficiency, while causing Ca and phosphorus (P) deficiencies and soil compaction, consequently reducing crop yield and quality [3,4,5]. Additionally, soil acidification degrades microbial habitats, inhibits their growth, and reduces biomass [6]. Studies have shown that soil acidification can be remediated by applying alkaline conditioners [7,8]. However, selecting appropriate soil conditioners requires comprehensive consideration of multiple factors, including soil test results, crop requirements, and environmental conditions, as well as the type and dosage of conditioners. Therefore, it is vital to find effective agents for alleviating soil acidification to improve agricultural production efficiency, ensure food security, enhance the ecological environment, and promote sustainable agricultural development.

Recent studies on improving acidic soils have primarily focused on the application of lime, biochar, and organic materials [9,10,11]. Lime-based soil conditioners can rapidly increase the pH levels in acidic soils, neutralize potential acidity, and reduce exchangeable acid (EA) ions, while supplementing Ca and magnesium. Commonly used lime materials include quicklime (CaO), slaked lime (Ca(OH)_2_), limestone (CaCO_3_), and dolomite powder (CaMg(CO_3_)_2_). However, the long-term use of lime may lead to soil compaction [10]. Although biochar shows significant potential for improving acidic soil and crop growth [11,12], its high cost remains a limitation, while the efficacy of organic materials in soil acidification remains controversial. Research indicates that decomposed straw matter effectively increases soil pH and alleviates acidity, consequently promoting maize growth [13]. However, in specific conditions, straw decomposition may increase the carbon dioxide and organic acids in the soil, which can accelerate the loss of base cations and exacerbate soil acidification [14]. These cases demonstrate that while these methods have improved soil properties and crop yields to some extent, they still present challenges, including high costs, limited resources, unclear improvement efficacy, complex acidification reversal processes, and environmental impacts [9,14]. Therefore, it is essential to continuously explore innovative technologies and develop more environmentally friendly and cost-effective improvement materials.

Phosphate tailings (PTs) are Ca-magnesium mineral aggregates formed during P ore processing, containing 30% to 40% of the total ore content [15]. Currently, most PTs are stored in tailing dams, which not only occupy land but also pose environmental risks such as heavy metal pollution [16]. PTs typically contain minerals such as dolomite, fluorapatite, and quartz. Notably, dolomite has been proven effective as an acidic soil conditioner [17,18], providing theoretical support for its application in soil improvement. PTs are rich in nutrients such as Ca, magnesium, and P, which enhance soil fertility by replenishing Ca ions, improving soil structure, increasing pH levels, and reducing the risk of ion imbalance [19]. Additionally, P supplementation promotes crop growth and dry matter accumulation in wheat [20]. During lead-contaminated soil remediation, thorough contact between the PTs and soil not only reduces lead bioavailability but also elevates soil pH [21]. The dissolution of its carbonate components helps neutralize acidity in acidic soils. The released Ca ions can effectively immobilize pollutants such as arsenic and antimony by forming insoluble Ca salts or enhancing the adsorption capacity, consequently reducing their bioavailability [22]. Although PTs have shown potential as conditioners for acidic soils, systematic studies involving their impact on soil quality, microbial community structure, and multi-cropping productivity remain limited. Therefore, it is crucial to comprehensively evaluate the integration of PTs as a soil amendment to develop this approach into an efficient, resource-efficient, and environmentally sustainable remediation strategy.

This study established a maize-potato rotation system in acidic dry red soil. Field trials were conducted using four PT application gradients to investigate their neutralizing effect on soil acidity, the evolution of physicochemical properties, the response characteristics of the microbial community, and the synergistic enhancement mechanism between crop yield and soil quality. The study proposes the following hypotheses: (1) PT application rapidly neutralizes soil acidity by releasing alkaline substances such as Ca and magnesium, improving soil quality to ensure crop yields in acidic soil rotation systems. (2) The improvement effect of PTs is dose-dependent, with the application frequency influencing their efficacy. (3) Specific PT-responsive microbial communities exist in acidic soils, where the community structure is closely related to soil productivity maintenance. Verifying these hypotheses helps to scientifically evaluate the practical efficacy of PTs in improving acidic soil. It also provides theoretical support for developing sustainable agricultural models based on the recycling and reuse of industrial and mining byproducts.

## 2. Results

### 2.1. Crop Yield and Soil Quality

**Figure 1:**
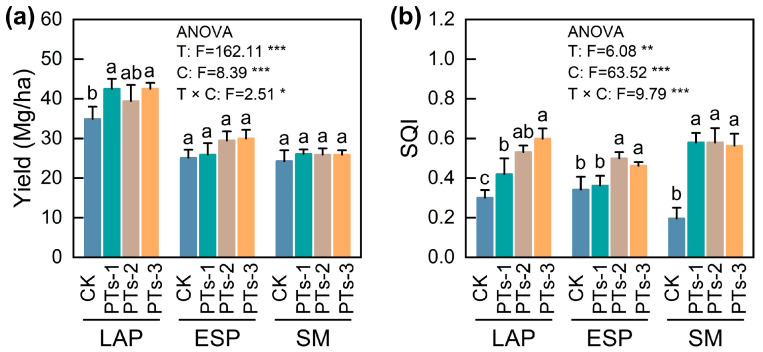
Changes in crop yield and soil quality under PT application. (**a**) Effects of PT application on crop yield. (**b**) Effects of PT application on soil quality. LAP, late autumn potato; ESP, early spring potato; SM, summer maize. T, different treatments; C, cropping system. Error bars represent the standard error of the mean (*n* = 4). Different lowercase letters indicate significant differences among treatments (*p* < 0.05). *, *p* < 0.05, **, *p* < 0.01 and ***, *p* < 0.001.

The PTs and multiple cropping rotation systems individually and interactively influenced the yield and soil quality of the crops (*p* < 0.05; Figure 1). Compared to the CK treatment, PT application significantly increased LAP yields, with PTs-1 and PTs-3 showing the most substantial increase at 21.67% and 21.82%, respectively (*p* < 0.05), while the ESP and SM displayed negligible yield trends (Figure 1a). Additionally, no significant differences were evident between the ESP yields after PTs-2 and PTs-3 treatment (Figure 1a). PT treatment also significantly improved the soil quality in the potato-maize rotation systems (*p* < 0.05; Figure 1b). Specifically, PTs-1, PTs-2, and PTs-3 treatment increased the soil quality during LAP cultivation by 39.43%, 76.51%, and 98.56%, respectively, compared to CK. However, during ESP cultivation, PTs-2 showed the highest increase at 46.23%, followed by PTs-3 at 34.97%, and PTs-1 at 6.03%. The PTs displayed an even more significant impact during SM cultivation, with PTs-1, PTs-2, and PTs-3 treatment increasing the soil quality by 195.81%, 195.65%, and 187.18%, respectively, compared to CK.

### 2.2. Physical, Chemical, and Biological Characteristics of the Soil

**Figure 2:**
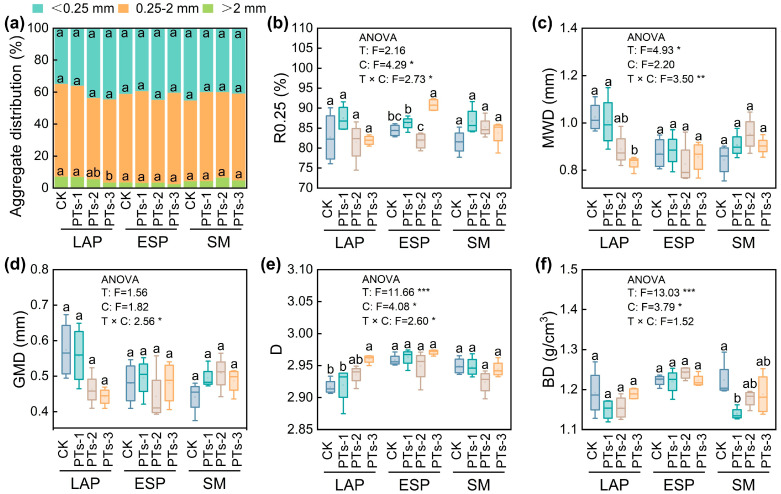
Changes in soil physical properties after PT application. (**a**) Distribution of soil aggregates under different phosphate tailings treatments. (**b**–**e**) Effects of phosphate tailings treatments on stability indices of water-stable aggregates. (**f**) Effects of phosphate tailings treatments on soil bulk density. LAP, late autumn potato; ESP, early spring potato; SM, summer maize. T, different treatments; C, cropping system. <0.25 mm: water-stable aggregates with particle size < 0.25 mm; 0.25–2 mm: water-stable aggregates with particle size between 0.25 and 2 mm; > 2 mm: water-stable aggregates with particle size > 2 mm; R0.25: content of aggregates > 0.25 mm; MWD: mean weight diameter; GMD: geometric mean diameter; D: fractal dimension; BD: soil bulk density. Error bars represent the standard error of the mean (*n* = 4). Different lowercase letters indicate significant differences among treatments (*p* < 0.05). *, *p* < 0.05, **, *p* < 0.01 and ***, *p* < 0.001.

**Figure 3:**
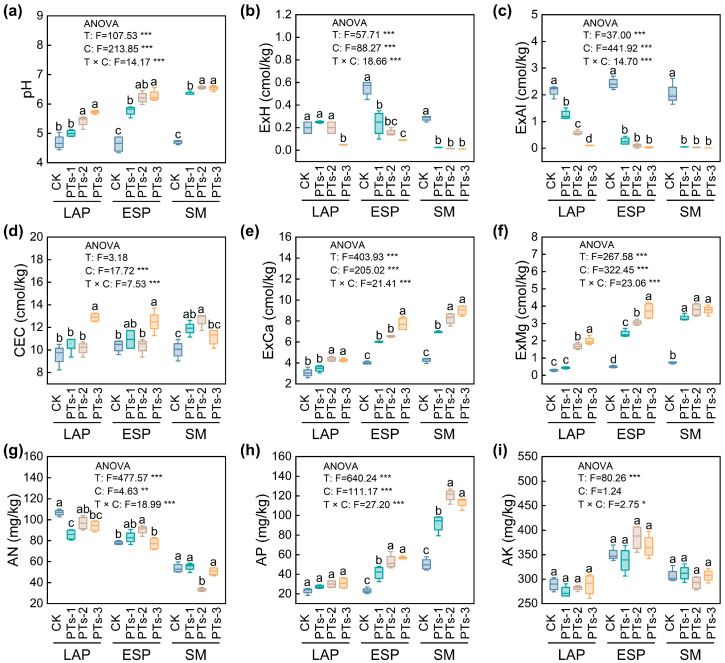
Changes in soil chemical properties after PT application. (**a**) pH: soil pH; (**b**) ExH: exchangeable hydrogen; (**c**) ExAl: exchangeable aluminum; (**d**) CEC: cation exchange capacity; (**e**) ExCa: exchangeable calcium; (**f**) ExMg: exchangeable magnesium; (**g**) AN: available nitrogen; (**h**) AP: available phosphorus; (**i**) AK: available potassium. LAP, late autumn potato; ESP, early spring potato; SM, summer maize. T, different treatments; C, cropping system. Error bars represent the standard error of the mean (*n* = 4). Different lowercase letters indicate significant differences among treatments (*p* < 0.05). *, *p* < 0.05, **, *p* < 0.01 and ***, *p* < 0.001.

**Figure 4:**
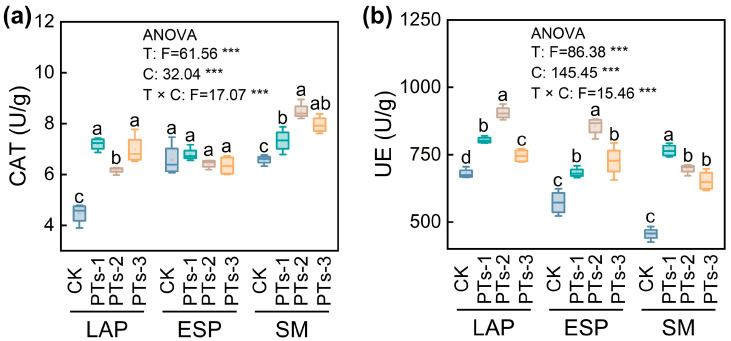
Changes in soil enzyme activities under PT application. (**a**) CAT: soil catalase activity; (**b**) UE: soil urease activity. LAP, late autumn potato; ESP, early spring potato; SM, summer maize. T, different treatments; C, cropping system. Error bars represent the standard deviation of the mean (*n* = 4). Different lowercase letters indicate significant differences among treatments (*p* < 0.05). ***, *p* < 0.001.

The PTs and multiple cropping rotation systems (*p* < 0.05; Figure 2, Figure 3 and Figure 4) significantly affected the physical, chemical, and biological properties of the soil. In potato-potato-maize rotation systems, the water-stable aggregates primarily consisted of small aggregates (0.25–2 mm), with microaggregates (<0.25 mm) being secondary (Figure 2a). PTs-3 treatment significantly reduced both the proportion and average weight diameter (MWD) of large aggregates (>2 mm) during LAP cultivation, which decreased by 51.12% and 18.89%, respectively, compared to the CK group (*p* < 0.05; Figure 2a,c), while its D increased by 1.49% (*p* < 0.05; Figure 2e). During ESP cultivation, PTs-3 treatment notably enhanced the mass fraction of aggregates > 0.25 mm (R_0.25_), which increased by 7.56% (*p* < 0.05; Figure 2b). During SM cultivation, the PTs-1 treatment significantly decreased the soil bulk density (BD) by 6.93%, compared to the CK group (*p* < 0.05; Figure 2f). No significant differences were evident between the GMD values across all treatments and cropping systems (Figure 2d).

PT treatment significantly increased the soil pH by 0.31–1.86 units, with the most substantial rise observed during ESP cultivation (1.13–1.64 units) (*p* < 0.05; Figure 3a). In the potato-potato-maize relay cropping system, PT treatment markedly enhanced the cation exchange capacity (CEC), ExCa, ExMg, and TEB (*p* < 0.05; Figure 3d–f and Figure S1d), while significantly reducing the EA, ExH, and ExAl (*p* < 0.05; Figure 3b,c and Figure S1a). During LAP cultivation, PT treatment significantly increased the exchangeable potassium (ExK) and TK but decreased the total nitrogen (TN) and AN levels (*p* < 0.05; Figure 3g and Figure S1b,f,h). Treatment during ESP cultivation significantly improved the ExK, exchangeable sodium (ExNa), TP, AN, and AP levels, but reduced the soil organic matter (SOM) (*p* < 0.05; Figure 3g,h and Figure S1b,c,e,g). SM cultivation demonstrated that treatment significantly increased the TP and AP content but decreased the AN (*p* < 0.05; Figure 3g,h and Figure S1g). No significant differences were evident between the available potassium (AK) after the various treatments (Figure 3i).

PT treatment significantly enhanced the soil catalase (CAT) activity, increasing from 38.48% to 61.32% in the LAP crop and from 11.47% to 29.00% in SM, where PTs-2 treatment demonstrated the highest efficacy (*p* < 0.05; Figure 4a). The soil urease (UE) activity also increased markedly after PT treatment (*p* < 0.05), with increases of 9.17–33.17% for LAP and 19.40–49.64% for ESP, where the PTs-2 treatment showed the greatest improvement. For SM, the increase ranged from 43.16% to 67.85%, with the PTs-1 treatment exhibiting the highest enhancement (Figure 4b).

### 2.3. Diversity, Composition, and Key Groups of the Bacterial and Fungal Communities in the Soil

**Figure 5:**
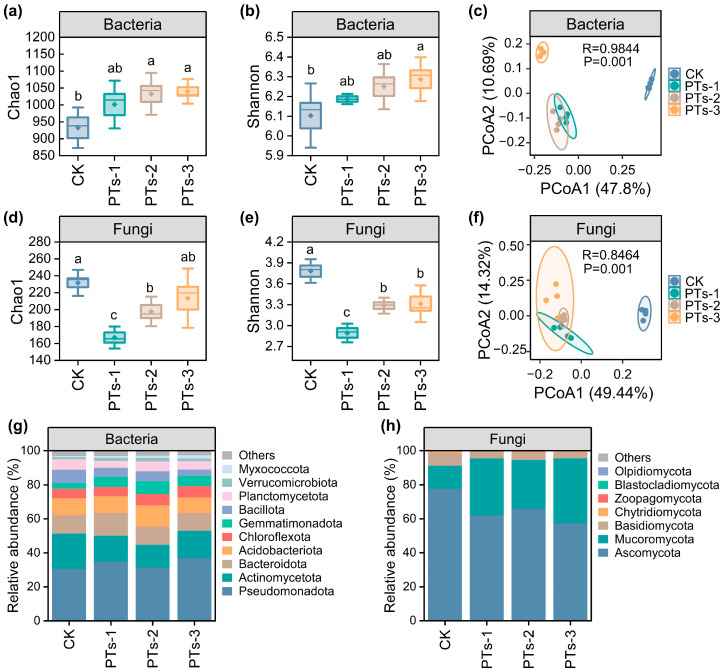
Changes in soil bacterial and fungal α-diversity, β-diversity, and community composition. (**a**,**b**) Bacterial community richness (Chao1 index) and diversity (Shannon index); (**d**,**e**) Fungal community richness (Chao1 index) and diversity (Shannon index); (**c**,**f**) Principal coordinate analysis (PCoA) of bacterial and fungal communities based on Bray–Curtis distance; (**g**,**h**) Composition of bacterial and fungal communities at the phylum level. Error bars represent the standard deviation of the mean (*n* = 4). The upper and lower edges of the box plots represent the 75th and 25th percentiles, respectively. The upper and lower whiskers indicate one standard deviation above and below the mean, respectively. Data points represent individual replicate samples. Different lowercase letters indicate significant differences among treatments (*p* < 0.05).

Further analysis of the microbial communities in the SM rhizosphere soil revealed that PT treatment significantly increased the bacterial α-diversity (Chao1 and Shannon indices) but decreased the fungal α-diversity (*p* < 0.05; Figure 5a,b,d,e). Principal coordinate analysis (PCoA) showed treatment-induced significant alterations in the bacterial (R = 0.98) and fungal (R = 0.85) community structures (*p* < 0.05; Figure 5c,f). At the phylum level, the bacterial communities in the soil were dominated by Pseudomonadota (33.75%), Actinomycetota (16.23%), Bacteroidota (11.36%), and Acidobacteriota (11.34%) (Figure 5g) while the fungal phyla primarily included Ascomycota (66.08%), Mucoromycota (28.26%), and Basidiomycota (5.10%) (Figure 5h). At the genus level, *Gemmatimonas* (5.45%), *Sphingomonas* (4.49%), and *Pseudarthrobacter* (3.58%) represented the dominant bacteria (Figure S2a), while the most abundant fungi included *Mortierella* (26.44%), *Humicola* (20.46%), and *Fusarium* (15.74%) (Figure S2b).

**Figure 6:**
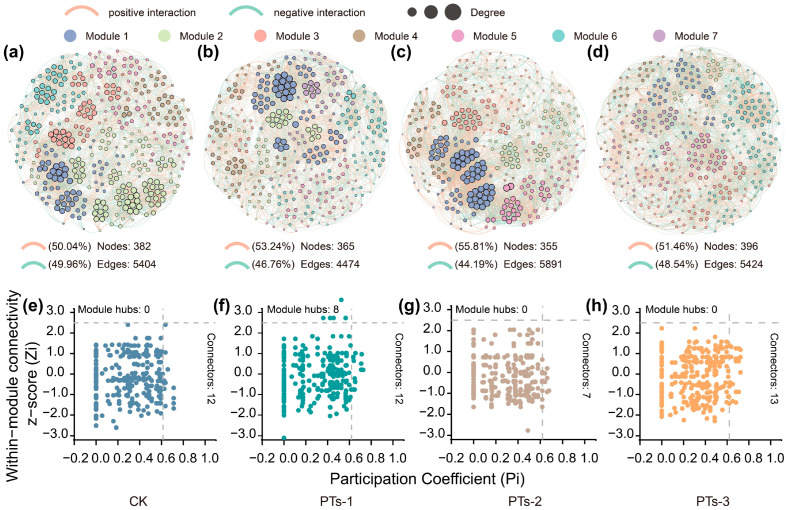
Bacterial-fungal co-occurrence networks and keystone taxa in the rhizosphere soil of maize under PT application. (**a**–**d**) Bacterial-fungal co-occurrence networks; (**e**–**h**) Keystone taxa in bacterial-fungal networks. Edges in light yellow and light green represent positive and negative connections, respectively. Modules within each network are colored differently. Nodes represent amplicon sequence variants (ASVs) of bacteria and fungi, with node size proportional to the number of connections (i.e., degree). Module hubs (Zi > 2.5 and Pi < 0.62), network hubs (Zi > 2.5 and Pi > 0.62), connectors (Zi < 2.5 and Pi > 0.62), and peripheral nodes (Zi < 2.5 and Pi < 0.62) are indicated.

In the bacterial co-occurrence network, the PTs-2 treatment group exhibited significantly higher positive-to-negative connection ratios (P/N), number of connections, average degree values, and average clustering coefficients compared to CK (Figure S3a–d; Table S1). Contrarily, the fungal networks showed an opposite trend, with the PTs-2 treatment group demonstrating significantly lower topological parameters, including node counts, connection edges, and average degree values, than the CK group (Figure S3e–h; Table S1). Notably, cross-species interactions between the bacteria and fungi also demonstrated the advantages of PTs-2 treatment. Marked improvements were evident in the positive-to-negative connection ratios, connection edges, average degree values, and average clustering coefficients (Figure 6a–d; Table S1). Zi-Pi analysis further identified 20, 7, and 13 keystone taxa in the PTs-1/PTs-2/PTs-3 treatment groups, respectively, compared to 12 keystone taxa in the CK group (Figure 6e–h and Figure S4b). The core network modules of all the treatment groups were concentrated in Modules 1–5 (Figure 6a–d). Correlation analysis confirmed significant associations between the microbial community composition and soil environmental factors (*p* < 0.05, Figure S5a). Positive correlations were evident with the key species distribution patterns and soil pH, ExCa, ExMg, TEB, TP, AP, and CAT concentrations (*p* < 0.05), while negative associations were apparent with the EA, ExH, and ExAl indicators (Figure S5b). These findings promote the understanding of the construction mechanism and ecological function of environmental microbial communities in PTs.

Bacterial connections are densest and most prevalent in microbial networks, while direct fungal linkages are relatively rare (Figure S4a). Compared to CK, PT treatment enhanced the bacterial interactions but reduced fungal contact, with PTs-2 treatment showing the most significant effect. The bacterial-fungal interactions exhibited an initial decline, followed by an increase (Figure S4a). PT treatment modified the key species composition of the cross-boundary networks (Figure 6e–h and Figure S4b), significantly increasing the abundance of key bacterial phyla such as Acidobacteriota +40.40–103.31% and Actinomycetota +25.30% to 37.80%, while reducing the abundance of Bacillota (−25.74% to −53.81%) and Bacteroidota (−13.22% to −33.26%) (Figure S4b). Among the fungi, the Ascomycota abundance increased by 18.11% to 48.30% (Figure S4b). In summary, continuous PT application differentially regulates the composition and coexistence network of the microbial communities in acidic soil, with these changes showing significant correlations with soil pH, EA, and other physicochemical properties (Figure S5).

### 2.4. Correlation Analysis Between the Soil Quality, Crop Yield, and Soil Properties

**Figure 7:**
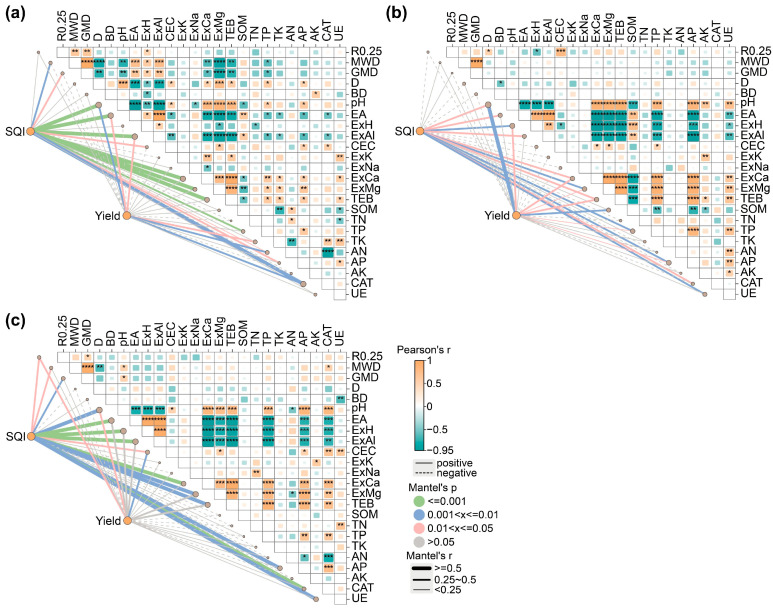
Correlations between soil quality, crop yield, and soil properties under PT application for (**a**) LAP, (**b**) ESP, and (**c**) SM. R0.25: mass percentage of soil aggregates > 0.25 mm; MWD: mean weight diameter; GMD: geometric mean diameter; D: fractal dimension; BD: bulk density; pH: soil pH; EA: exchangeable acidity; ExH: exchangeable hydrogen; ExAl: exchangeable aluminum; CEC: cation exchange capacity; ExK: exchangeable potassium; ExNa: exchangeable sodium; ExCa: exchangeable calcium; ExMg: exchangeable magnesium; TEB: total exchangeable bases; SOM: soil organic matter; TN: total nitrogen; TP: total phosphorus; TK: total potassium; AN: available nitrogen; AP: available phosphorus; AK: available potassium; SCAT: soil catalase activity; SUE: soil urease activity. Correlations between soil quality, crop yield, and various factors were determined by Mantel tests based on Bray–Curtis distance. Edge width corresponds to Mantel’s r statistic, and edge color indicates statistical significance based on 999 permutation tests. The color gradient represents Spearman’s correlation coefficients for pairwise correlations between factors. *, *p* < 0.05, **, *p* < 0.01, ***, *p* < 0.001, and ****, *p* < 0.0001.

A Mantel test revealed correlations between the soil quality, crop yield, and soil properties (Figure 7). In the potato-potato-maize relay cropping systems, both the soil quality and yields were significantly associated with pH (Figure 7, *p* < 0.05). During LAP cultivation, the soil quality and yields were also significantly correlated with EA, ExAl, AN, and CAT (Figure 7a, *p* < 0.05). ESP cultivation exhibited significant correlations with ExH, ExCa, ExMg, TEB, TP, and AP (Figure 7b, *p* < 0.05). R0.25, CEC, and Module 5 represented the relevant factors in the SM cropping systems (Figure 7c, *p* < 0.05).

### 2.5. Determinants of Crop Yield

Linear regression analysis demonstrated that the LAP and ESP yields showed significantly positive correlations with soil quality (*p* < 0.05, Figure S6a,b), while SM production exhibited no correlation with soil quality (Figure S6c). Furthermore, random forest model analysis revealed that pH, EA, ExAl, ExCa, ExMg, and TP were key factors influencing soil quality (*p* < 0.05; Figure S7).

**Figure 8:**
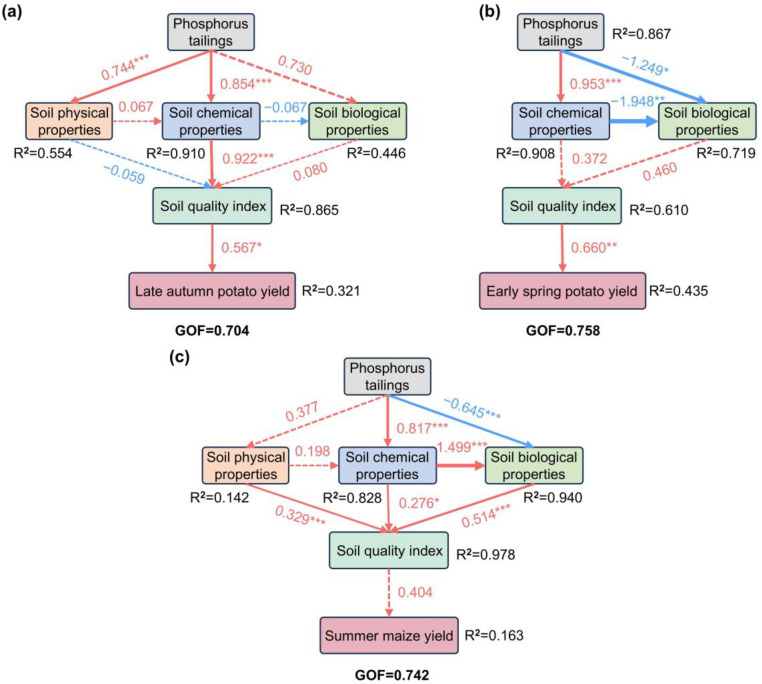
Partial least squares path modeling (PLS-PM) analysis revealing the effects of soil properties on the yield of (**a**) LAP, (**b**) ESP, and (**c**) SM. Solid and dashed arrows represent significant positive and non-significant path coefficients, respectively, with a *p*-value threshold of 0.05. Numbers adjacent to arrows indicate standardized path coefficients. Numbers within boxes represent loading factors between latent (soil physical, chemical, and biological properties) and observed variables. Line width is proportional to the coefficient magnitude. R^2^ values represent the proportion of variance in crop yield explained by the model. GOF, goodness of fit. Key taxa and network modules are represented by the first principal coordinate (PCo1) values. *, *p* < 0.05, **, *p* < 0.01 and ***, *p* < 0.001.

Partial least squares path modeling (PLS-PM) analysis demonstrated that in AP cultivation, PTs indirectly improved soil quality and ultimately increased potato yield by directly regulating soil chemical properties (pH, ExH, ExAl, EA, ExCa, ExMg, TEB, and TP) and aggregate structure stability (MWD, D) (*p* < 0.05; Figure 8a). Although the PTs did not have a significant direct impact on the soil quality, they substantially influenced the chemical properties, including pH, ExAl, EA, ExCa, ExMg, SOM, TP, and AP, as well as biological properties, such as UE, in the soil. The effect of soil quality was signified by a higher ESP yield (*p* < 0.05; Figure 8b). Additionally, a significant positive correlation was apparent between the chemical properties in the soil and UE (*p* < 0.01; Figure 8b). Similarly, the PTs directly affected soil chemical properties (pH, ExH, ExAl, EA, ExCa, ExMg, TEB, TP, AP) and biological properties (Module 1/3/4/5, Keystone, and UE) (*p* < 0.05; Figure 8c), while indirectly improving the soil quality. However, the soil quality did not significantly enhance the yield. The biological properties in the soil were regulated by the chemical properties (*p* < 0.05; Figure 8c), while the aggregate structure (MWD and GMD) directly influenced the soil quality (*p* < 0.05; Figure 8c).

## 3. Discussion

This study investigated the effect of PTs on the properties and microbial community composition in acidic soil in maize-potato rotation systems, as well as their impact on improving soil quality and crop yield (maize and potato). Employing PTs for soil improvement represents a sustainable resource utilization approach that helps reduce the environmental impact of industrial waste. Unlike traditional soil amendment studies, this research not only systematically evaluated the environmental impact of PTs but also revealed the dose–response relationship between their application rates and their dependence on application frequency. Additionally, this research further analyzed the microbial community structure responses to continuous PT application and identified key microbial groups with potential functions during nutrient cycling. The results demonstrated that two consecutive applications of 9.0 t·ha^−1^ (PTs-2) PTs outperformed other treatments in enhancing soil quality and crop yields. In terms of the microbial community structure, this treatment induced notable variations in the diversity of the rhizosphere bacteria and fungi compared to CK (Figure 1 and Figure 5). Rhizosphere microbial communities, often referred to as the plant’s “second genome,” are crucial for promoting plant growth and nutrient uptake, and controlling disease. However, soil acidification frequently disrupts the ecological balance and functional processes of microbial communities [23]. The findings indicated that PTs improved soil quality and crop yields by modifying the chemical properties of acidic soils (e.g., increasing pH levels and Ca/Mg content), while regulating the microbial community structure and functions.

### 3.1. The Effect of PTs on the Soil Properties

Varying PT application rates significantly affected the physical soil structure in the maize-potato rotation systems. Soil aggregate analysis revealed that the system predominantly contained small 0.25–2 mm water-stable aggregates, with higher proportions than < 0.25 mm microaggregates (Figure 2a), which was consistent with findings by Yadav et al. [24] in other rotation systems. Although PTs-3 treatment rapidly neutralized soil acidity, it reduced aggregate stability. The proportion of large aggregates >2 mm decreased in the LAP crops, showing a minimum MWD and maximum D (Figure 2a,c,e), while the mass fraction of the aggregates > 0.25 mm increased significantly in the ESP crops (Figure 2b). Dai et al. [25] noted that intensive tillage sheared large aggregates, which was consistent with the results of this study, showing that excessive PTs caused the disintegration of aggregates > 2 mm. This may be attributed to improper binding between high-dose PT particles and soil colloids, or because their rapid ion release disrupts the soil particle bonding equilibrium. Conversely, the Ca^2+^ ions released by the dolomite and calcite in PTs promote the formation of small 0.25–0.5 mm aggregates [26], explaining the simultaneous R_0.25_ increase and MWD decrease. Lower aggregate stability may also reduce the carbon sequestration and erosion resistance in the soil. Dai et al. [25] revealed that the soil erosion modulus increased by 15–20% for every 0.1 mm MWD decrease. Therefore, since excessive PTs may reduce agglutinate stability, the soil carbon sequestration potential, and erosion resistance, the short-term improvement effect and long-term soil risk should be considered.

Maintaining optimal pH levels is crucial for sustaining the biological activity in soil [25]. In this study, PT treatment increased the soil pH by 0.31–1.86 units compared to CK, with the most significant enhancement observed during ESP cultivation (Figure 3a). The pH increase was primarily attributed to three mechanisms: the neutralization of H^+^ by the OH^−^ and CO_3_^2−^ released via dolomite and calcite dissolution, the conversion of HPO_4_^2−^ into H_2_PO_4_^−^ via proton consumption, and Al^3+^ precipitation into Al(OH)_3_ hydroxide through the ion exchange mediated by Ca^2+^/Mg^2+^ [27,28,29,30]. The sustained pH elevation throughout the crop rotation cycle confirmed the controlled-release improvement properties of the PTs. This process evolved through two initial stages: the slow dissolution of dolomite and calcite minerals in the acidic soils during the early treatment, followed by accelerated mineral weathering driven by increased organic acid secretion from the crop roots and microbial metabolism in later stages. This process continuously released OH^−^ and alkaline cations, consequently maintaining a stable pH level. PTs significantly enhanced the CEC, ExCa, ExMg, and TEB (Figure 3d–f and Figure S1d), while reducing the EA, ExH, and ExAl (Figure 3b,c and Figure S1a). This demonstrated their combined efficacy in improving acid soils via calcium and magnesium release, proton consumption, and aluminum precipitation. The TK levels increased significantly in the AP fields (Figure S1h), which was attributed to the slow dissolution and release of potassium minerals in the PTs [31]. Continuous PT application also markedly improved the TP and AP levels in the soil during ESP and SM (Figure 3h and Figure S1g). This was attributed to two mechanisms: the direct provision of P_2_O_5_ and the activation of phosphorus-soluble bacteria (PSBs) that enhanced the *p* potential in the soil. PSBs secrete organic acids to dissolve inorganic *p*, which are then degraded by *phoD* gene-encoded acid phosphatases. This synergistic action of inorganic and organic P dissolution improves P availability [32].

CAT and UE are key enzymes that characterize the biological activity and health status of soil. CAT alleviates oxidative stress by degrading the H_2_O_2_ in soil, which protects microbial cell membrane integrity and indirectly maintains soil respiration functions [33]. UE catalyzes urea hydrolysis into ammonium nitrogen, reducing ammonia volatilization and enhancing nitrogen utilization efficiency [34]. This study demonstrated that PTs significantly enhanced CAT and UE activity, with UE showing the most substantial increase in the biotite-rich potato plants, peaking after PTs-2 treatment (Figure 4a,b). The enhanced enzyme activity was ascribed to both direct and indirect mechanisms: Ca^2+^/Mg^2+^ stimulated the PSB secretion of organic acids, upregulated *katE* and *ureC* gene expression, and promoted enzymatic synthesis [34,35]. Furthermore, pH neutralization mitigated the H^+^ inhibition of enzyme active centers [36]. These findings indicated that PTs synergistically enhanced soil enzyme functionality by promoting mineral dissolution, microbial activation, and pH elevation adaptation.

### 3.2. Bacterial and Fungal Response to the PTs During Acidic Soil Remediation

This study investigated the effect of the continuous application of different PTs dosages on the microbial community structures in the soil. The results showed that PTs significantly increased the Chao1 and Shannon indices of the soil bacteria (Figure 5a,b). Bacterial diversity is generally low in acidic soils (pH < 5.5), particularly at pH < 5, with a marked decline in the Shannon index. This is mainly because high H^+^ and Al^3+^ concentrations inhibit dominant functional bacterial groups such as Acidobacteria and Pseudomonadota [23,37]. In this study, the PTs neutralized soil acidity (increasing pH from 4.67 to 0.31–1.86 units), effectively eliminating this inhibitory effect and significantly enhancing the relative abundance of Acidobacteria and Pseudomonadota (Figure 5g). Conversely, the PTs substantially reduced the fungal Chao1 and Shannon indices (Figure 5d,e), likely due to the enrichment of beneficial fungi such as *Mortierella* (relative abundance 26.44%). These fungi promote plant growth through P dissolution, immune induction, and microbial network regulation [38,39]. PCoA further showed that PT treatment significantly altered the bacterial and fungal community structures (Figure 5c,f). In conclusion, PTs promoted plant growth by enhancing bacterial diversity and regulating fungal composition.

The complexity of microbial co-occurrence networks is closely related to soil multifunctionality [40,41]. This study found that the topological parameters (e.g., node count, connectivity, and modularity) of the bacterial-bacterial, fungal-fungal, and cross-boundary networks were superior to those in CK after PT treatment (Figure 6a–d, Figure S3 and Figure S4; Table S1), indicating more complex network structures and higher stability. Highly modular networks, such as the functional zones formed by saprophytic fungi and P-oxidizing bacteria, can accelerate carbon-P transformation via interspecies synergy. Additionally, a high positive-to-negative connectivity ratio (P/N, where P represents positive synergistic connections and N denotes negative competitive/inhibitory connections) and average degree (the number of connections per species) further enhance network functionality, improving redundancy and disturbance resistance. Notably, PT treatment significantly increased the relative abundance of bacterial phyla (Acidobacteriota and Actinomycetota) and fungal ascomycetes, while reducing that of thick-walled bacteria (Bacillota) and Bacteroidota, likely due to their acid tolerance and pH-sensitive growth patterns. The phosphorellic acid-oxidizing bacteria in the Actinobacteria phylum dissolve soil insoluble P via organic acid secretion, while the saprophytic fungi in the Ascomycota phylum transport dissolved P to plant roots along extended mycelial networks. This forms a synergistic functional module of “bacterial P dissolution-fungal P transport,” which significantly enhances P utilization efficiency [42,43]. The network modularity increased from 0.42 to 0.58, further demonstrating the transformation of the soil microbial system from “inefficient competition” to “efficient collaboration,” markedly improving the ecological function stability and resilience.

### 3.3. The Impact of Soil Properties and Soil Quality on Crop Yield

While research on the utilization of PTs to improve acidic soil remains limited, their alkaline properties and nutrient-rich characteristics demonstrate potential for mitigating soil acidity, enhancing nutrient availability, and improving crop yields [44,45]. This study showed that two consecutive applications of 9.0 t·ha^−1^ PTs significantly reduced soil acidity and improved the physical properties and nutrient availability. This enhanced the soil quality and increased maize and potato yields, which were consistent with Hypotheses (1) and (2). Soil acidification typically leads to deficiencies in critical elements such as Ca and P, severely limiting crop growth and causing yield losses [5,11,46]. However, the excessive application of PTs may adversely affect the soil structure.

The interaction between PTs, soil properties, soil quality, and crop yields remains unclear and requires further investigation. The PLS-PM has preliminarily revealed the potential mechanisms. The results indicate that PTs not only directly improve soil properties but also indirectly enhance maize and potato yields by elevating soil quality (Figure 8). Further analysis showed significant correlations between the pH, SQI, and crop yields. The pH level significantly influenced the soil quality (Figure 7 and Figure S7), highlighting its core role in agricultural ecosystems. Soil pH directly regulates the solubility and adsorption behavior of nutrients. In acidic conditions (pH < 5.5), aluminum toxicity (Al^3+^) inhibits root growth and nutrient uptake [47]. Furthermore, pH changes drive microbial functional community succession. For instance, a pH below 5.5 suppresses nitrifying bacterial activity, which hinders nitrogen mineralization and exacerbates yield fluctuations [48,49]. This study also confirmed that the soil pH significantly affected bacterial and fungal community structures (Figure S5b). Particularly during ESP and SM seasons, the chemical properties of the soil positively influenced the biological characteristics (Figure 8b,c). In SM, certain network modules (Modules 1, 3, 4, and 5) and key microbial groups showed a positive correlation with the pH level and CAT activity, while a negative association was evident with EA, ExH, and ExAl (Figure S5b). Therefore, it is inferred that PTs may indirectly improve soil quality by regulating pH, which affects key microbial functions such as P-solubilizing bacterial proliferation and CAT secretion.

In summary, the PLS-PM model results indicated that soil properties represented the dominant factors influencing soil quality and crop yield, with key bacterial and fungal communities playing significant mediating roles. Appropriate PT application effectively improved the structure and functionality of acidic soils while enhancing crop productivity. Although a preliminary understanding has been gained regarding the functions of these critical microorganisms, future research should further isolate and identify key functional microorganisms (such as Actinomycetota and *Mortierella*), verify their synergistic interaction with PTs via pure culture experiments, and explore a combined PTs+functional microbial agent improvement model. By optimizing fertilization practices, this approach will provide more operational technical strategies for the sustainable improvement of acidic soils.

The detection results of this study indicate that the heavy metal content in the utilized PTs samples is below the relevant limit standards, providing a preliminary safety basis for their resource utilization in the field of environmental remediation. However, it is essential to recognize the dualism in the environmental behavior of PTs towards heavy metals. On one hand, studies have reported that PTs can effectively immobilize heavy metals such as arsenic, antimony, and lead in soil, demonstrating significant potential as materials for pollution remediation [21,22]. On the other hand, this immobilization capability stems precisely from their specific chemical composition, which is highly dependent on the origin of the raw phosphate ore, resulting in significant heterogeneity in the heavy metal content across different batches of PTs. Consequently, PTs from certain sources may themselves become sources of heavy metal pollution, as indicated by the environmental risks associated with tailing ponds cited in this study. This dual role as both a “remediating agent” and a “potential pollutant” dictates that conducting a rigorous analysis of the heavy metal content for any specific source of PTs prior to practical application is absolutely imperative. This is the only way to mitigate environmental risks and ensure their safe resource utilization.

## 4. Materials and Methods

### 4.1. Overview of the Experimental Site

The experimental site is characterized by mountainous red soil and is located in Luliang County, Qujing City, Yunnan Province (N 23°26′22″, E 103°2′41″), at an altitude of 1870 m with a subtropical plateau monsoon climate. The region has an annual average temperature of 15 °C and annual precipitation ranging between 960 mm and 1200 mm, with rainfall concentrated between July and September. The basic soil properties are given in Table 1 (analytical methods for these properties are described in Section 4.3). Yunnan Phosphorus Chemical Group Co., Ltd (Kunming, China). provided the PTs, which contained 5.67% P, 29.14% CaO, 12.26% MgO, 4.78% SiO_2_, and 0.76% Fe_2_O_3_, with a pH of 8.3. Hg, As, Pb, Cd, and Cr were present at concentrations of 0.2 mg·kg^−1^, 12.5 mg·kg^−1^, 49.6 mg·kg^−1^, 0.03 mg·kg^−1^, and 21.0 mg·kg^−1^, respectively, all meeting the heavy metal limit standards for soil conditioners. The PTs are dried, ground, passed through a 2 mm screen, and sealed for storage.

### 4.2. Test Design and Sample Collection

**Figure 9:**
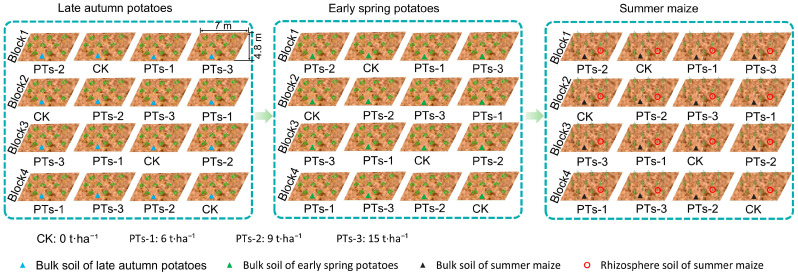
Diagram of sampling sites for the different treatments in the field experiments.

The experiment employed a randomized block design with four treatment strategies: the control (CK, 0 t·ha^−1^) and three PT treatments (PTs-1, 6 t·ha^−1^; PTs-2, 9 t·ha^−1^; and PTs-3, 15 t·ha^−1^), each replicated four times to form 16 plots (33.6 m^2^, 4.8 m × 7 m) (Figure 9). Crop rotations were implemented from 14 August to 9 December 2023, for the LAP (Lishu No. 6), 18 January to 18 May 2024, for the ESP (Deshu No. 7), and 22 May to 28 August 2024, for the SM (Guangliangtian No. 27). The potato rows and plants were spaced 25 cm apart, totaling 189 plants per plot with seven rows. The maize rows and plants were spaced 25 cm apart, totaling 162 plants per plot with six rows. A 2 m-wide protective row was established around each plot. PT powder was evenly distributed on the soil surface and incorporated during plowing. Both the potatoes and maize received basal and top-dressing fertilization. The LAP cultivation required a single basal fertilizer application before sowing, containing 81 kg/ha of pure urea (N, 46%), 90 kg·ha^−1^ of superphosphate (P, 12%), and 105 kg·ha^−1^ of potassium oxide (K, 51%). The first top-dressing was administered during tuber formation (54 kg·ha^−1^ N and 45 kg·ha^−1^ K), followed by a second top-dressing during tuber enlargement (30 kg·ha^−1^ N and 30 kg·ha^−1^ K). ESP cultivation involved a single basal fertilizer application before sowing, containing 60 kg·ha^−1^ N, 180 kg·ha^−1^ P, 180 kg·ha^−1^ K, 15 kg·ha^−1^ Na_2_B_4_O_7_.10H_2_0, and 15 kg·ha^−1^ ZnSO_4_.7H_2_O. The first top-dressing was applied during tuber formation (105 kg·ha^−1^ N and 120 kg·ha^−1^ K), followed by a second top-dressing during tuber enlargement (60 kg·ha^−1^ N and 75 kg·ha^−1^ K). The SM cultivation involved one application of a basal fertilizer before sowing, which contained 90 kg·ha^−1^ of N, 150 kg·ha^−1^ of P, and 90 kg·ha^−1^ of K. The first top-dressing was administered during the jointing stage (90 kg·ha^−1^ N and 60 kg·ha^−1^ K), followed by a second top-dressing at the large trumpet stage (120 kg·ha^−1^ N). Both the potato and maize crops underwent consistent field management practices, including weeding and pesticide spraying throughout their growth cycles. Identical management protocols were employed for all the experimental plots.

Three central rows were selected from each plot to measure the yield during potato harvesting, which was converted into the total plot yield and further adjusted to the unit area yield. For SM harvesting, four intact rows in the center of each plot were chosen to measure the fresh grain weight, with yields calculated per unit area. Five maize plants were randomly selected from each plot for harvesting. The entire plant was removed, and the loose soil was gently shaken off. The soil that tightly adhered to the roots was collected using sterile brushes to obtain rhizosphere soil samples. These five samples were combined into a single plot sample (a total of 16 samples), passed through a 2 mm sterile soil sieve, and then transferred to a sterile centrifuge tube on ice before being processed in the laboratory. Some samples were immediately used for soil enzyme activity assays, while the remainder was rapidly stored at −80 °C for subsequent microbial community sequencing. The five-point sampling method was used to collect surface soil from the LAP, ESP, and SM plots at depths ranging from 0 cm to 20 cm. The soil samples were passed through an 8 mm mesh to remove visible impurities, mixed, air-dried, and then sieved to a particle size of ≤2 mm for determination of SOC, TP, total potassium (TK), AP, pH, and ExCa levels. Intact soil was collected using a ring-knife sampler to assess the water-stable aggregate composition. Care was taken to avoid disturbance during collection and transportation to preserve the integrity of the aggregate structure. After natural drying, the quartering method was employed to divide the intact soil into 500 g portions for later use.

### 4.3. Analysis of the Soil Sample Properties

A combined dry-screen and wet-screen method was employed to determine the water-stable aggregates. Naturally air-dried soil, devoid of stones and plant residues larger than 5 mm in diameter, was placed on a 20 cm sieve stack with progressively finer screens of 5 mm, 2 mm, 1 mm, 0.5 mm, 0.25 mm, and 0.106 mm. After manual screening for 5 min, aggregates of each size were collected and weighed to calculate their mass percentages based on the dry-screen analysis. These fractions were proportionally blended into a 50 g soil sample, which was pre-moistened for 15 min and fed into the upper layer of the wet-screen device with 2 mm, 1 mm, 0.5 mm, 0.25 mm, and 0.106 mm screens. The instrument was activated at 30 vibrations per minute for 15 min of continuous wet screening, while ensuring that the water level covered the upper screen layer without overflow. Residual aggregates from each sieve layer were transferred to aluminum containers, air-dried at 105 °C until reaching a constant weight, and weighed to determine their mass percentages. The soil masses passing through the 2 mm and 0.25 mm screens were classified into three aggregate size grades: large aggregates (lma > 2 mm), small aggregates (sma 0.25–2 mm), and microaggregates (mia < 0.25 mm) [50,51]. The soil bulk density (BD) was measured using a 100 cm^3^ ring knife as specified in Soil Agricultural Chemistry Analysis [52].

The soil pH (soil/water = 1:2.5) was determined using a pH meter (Metro-pH 320; Mettler-Toledo Instruments Ltd., Shanghai, China) [52]. The EA and ExAl were measured using 1 mol·L^−1^ KCl exchange-neutralization titration, while the CEC was determined via 1 mol·L^−1^ ammonium acetate exchange [52,53]. The ExK and sodium (ExK and ExNa) were measured using ammonium acetate exchange-flame photometry, while the ExCa and ExMg were determined via 1 mol·L^−1^ ammonium acetate exchange-atomic absorption spectrophotometry [54]. The soil organic matter was measured via the K_2_Cr_2_O_7_ volumetric technique (external heating) while the TN was determined using the semi-micro Kjeldahl method [52,55]. The TP and TK were measured via the NaOH molybdenum antimony chromogenic method and NaOH molybdenum antimony flame photometry, respectively [52,55]. The AN was measured using alkali decomposition diffusion, while the Available P was determined using a 0.5 mol·L^−1^ HCl-0.025 mol·L^−1^ (1/2 H_2_SO_4_) solution [52,55]. The AK was measured via NH_4_OAc leaching-flame photometry [52,55]. The TEB = ExK + ExNa + ExCa + ExMg.

Soil urease (UE) activity was determined by the indophenol blue method, which measures the ammonium released from urea after incubating the soil at 37 °C for 24 h. The absorbance of the resulting solution was read at 690 nm using a SpectraMax iD3 microplate reader. Soil catalase (CAT) activity was measured by the potassium permanganate titration method, which quantifies the decomposition of hydrogen peroxide after a fixed reaction time. All assays were performed following the manufacturer’s protocols (Soil Enzyme Assay Kit, Beijing Boxbio Science & Technology Co., Ltd., Beijing, China, Catalog No: AKEN023M-100S (UE), AKEN001M-100S (CAT)).

### 4.4. 16S rDNA and ITS rDNA Sequencing and Bioinformatics Analysis

An E.Z.N.A@ Soil DNA Extraction Kit (Omega Biotek, Norcross, GA, USA) was used to extract the genomic DNA from each soil sample. The PCR amplification of the bacterial 16S rDNA V1-V9 and fungal ITS1-ITS4 regions was performed using the 27F (5′-AGRGTTYGATYMTGGCTCAG-3′)/1492R (5′-RGYTACCTTGTTACGACTT-3′) and ITS1F (5′-CTTGGTCATTTAGAGGAAGTAA-3′)/ITS4R (5′-TCCTCCGCTTATTGATATGC-3′) primers, respectively. The 20 µL PCR reaction system contained 4 µL 5× FastPfu buffer, 5 µM forward and reverse primers, 2.5 mM dNTPs, 0.4 µL FastPfu polymerase, and 10 ng template DNA. The amplified products were purified using an AxyPrep DNA Gel Extraction Kit (Axygen Biosciences, Union City, CA, USA) and quantified via PCR using a Qubit R 3.0 analyzer (Thermo Fisher Scientific, Waltham, MA, USA). The amplified libraries were sequenced on the Illumina MiSeq PE250 platform (Biozeron Biological Technology Co., Ltd., Shanghai, China) according to standard protocols (http://www.illumina.com (accessed on 21 February 2025)).

After sequencing, the raw fastq data were analyzed using the QIIME2 algorithm (https://qiime2.org/ (accessed on 21 February 2025)) to remove the primer and index barcodes, followed by merging into paired-end sequences. After quality control, noise removal, and chimeric sequence elimination, the optimized bacterial and fungal ASVs were clustered against the SILVA database (Release 138) and UNITE v7.2 (Full UNITE + INSD datasets) using a 97% similarity threshold. Samples containing single-specific ASVs were excluded, while the lowest read counts across all samples were used for normalization. Sequencing results were submitted to the Sequence Read Archive at the National Centre for Biotechnology Information under Bioproject PRJNA1328184.

The “vegan” package in R 4.1.0 was used to analyze the α-diversity and β-richness of the bacterial and fungal communities. PERMANOVA analysis was employed to identify the significant differences between the microbial community structures in the maize rhizosphere soil after the various treatments. A Mantel test was used to evaluate the correlation between bacterial and fungal community distance matrices (UniFrac distance matrices) and the environmental variable distance matrices.

Cross-species network analysis was employed to assess the interactions between the bacterial and fungal communities [56]. Correlation matrices (r > 0.7, *p* < 0.05) were constructed using the “igraph” (version 2.0.3) and “Hmisc” (version 5.2-2) software packages to create co-connection networks. The final network was visualized using Gephi 0.10.1, while the topological features, including the connection number (number of edges between the nodes), positive/negative connection ratio, average degree, average path length, clustering coefficient, and modularity (higher values indicate stronger connectivity), were calculated [57]. The characteristic module genes were identified as the first principal component of the standardized relative abundance matrices of the ASVs within the modules. The node roles were classified based on inter-module (Pi) and intra-module connectivity (Zi), combined with the average node degree [58,59]. The nodes were categorized into five types: module hubs (Zi > 2.5), network hubs (Zi > 2.5 and Pi > 0.62), connectors (Pi > 0.62), and edge nodes (Zi < 2.5 and Pi < 0.62). The module hubs, network hubs, and connectors were defined as keystone taxa.

### 4.5. Characteristic Hydrolytic Stability Index and Soil Quality Index (SQI) Analysis

#### 4.5.1. Calculation of the Characteristic Soil Water Aggregate Stability Index

The soil aggregates were evaluated by examining the mass fraction (R_0.25_), mean weight diameter (MWD), geometric mean diameter (GMD), and fractal dimension (D) of >0.25 mm aggregates. The calculation formula was as follows:

(1) $$R_{0.25}=1-\;\frac{M_{x\;<\;0.25}}{M_{T}}$$

(2) $$\mathrm{MWD}=\sum_{i=1}^{n}x_{i}\;\times \;W_{i}$$

(3) $$\mathrm{GMD}=\exp\left(\frac{\Sigma_{\mathrm{i}=1}^{n}m_{i}\ln R_{i}}{\Sigma_{\mathrm{i}=1}^{n}m_{i}}\right)$$

(4) $$\frac{M\left(r<{\bar{R}}_{i}\right)}{M_{T}}={\left(\frac{{\bar{R}}_{i}}{R_{max}}\right)}^{3-D}$$

In this formula, n represents the number of particle size groups, *x_i_* denotes the average diameter of the i-th particle size group aggregate, *W_i_* indicates the mass of the i-th size group aggregate, *Mx* < 0.25 signifies the mass of aggregates with diameters smaller than 0.25 mm, *M_T_* is the total mass of all the aggregates, *R_max_* is the average diameter of the largest aggregate, and R_0.25_ refers to the mass fraction of mechanically stable aggregates larger than 0.25 mm.

#### 4.5.2. SQI Calculation

The SQI was established via principal component analysis (PCA) using a method delineated by Li et al. [60] to determine the physicochemical properties and enzyme activity indicators in the soil. A minimum data set (MDS) was constructed to quantify the SQI and evaluate the extent to which treatment improved the soil quality. After scoring and weighting the MDS indicators, the SQI was defined as:

(5) $$\mathrm{SQI}=\sum_{i=1}^{n}W\;\times \;S_{i}$$

(6) $$Q_{\mathrm{SQI}}=\sum_{i=1}^{n}WiS_{i}$$

In this formula, *Wi* is the PC weighting factor, and *Si* denotes the soil score of variable *i*.

### 4.6. Statistical Analysis

Two-way ANOVA was combined with Duncan’s post hoc test (*p* < 0.05) to evaluate the impact of the cultivation systems on the physical, chemical, and biological properties, as well as other response variables in the soil. Mantel tests were employed to analyze the correlations between the soil quality, crop yield, and physicochemical and biological characteristics. A random forest model was constructed using the “RandomForest” package to identify the key factors influencing soil quality. The importance of each factor was determined by assessing the degree of prediction accuracy decline or the mean squared error (MSE) increment between the observed and predicted values. The model and predictor significance were calculated using the “rfUtilities” and “rfPermute” packages, respectively. The significant predictors identified via the random forest analysis were used for subsequent partial least squares path modeling (PLS-PM) to investigate the direct and indirect impact of the chemical, physical, and biological properties in the soil on the crop yield. The PLS-PM model was constructed using the plspm package, and variables with loadings below 0.5 were excluded. The final model was constructed under the hypothesis that PT input affected crop yield by regulating soil physics, chemistry, enzymatic activity, and microorganisms. Model fit was evaluated using the goodness-of-fit index (GOF), with a threshold of 0.40 < GOF ≤ 1.00 considered acceptable [61].

## 5. Conclusions

This study demonstrated that moderate PT application effectively improved acidic red soil. It enhanced soil quality and solid waste resource utilization, while simultaneously improving the crop yields in maize-potato rotation systems. The efficacy of PTs in improving the physical, chemical, and biological characteristics of soil exhibited a significant dose-dependent effect. The continuous two-treatment application of 9.0 t·ha^−1^ PTs (PTs-2 treatment) substantially improved acidic red soil, increasing the SQI by 46.23% compared to CK. This was primarily because PTs neutralized soil acidity, which optimized the chemical properties and enhanced the biological characteristics. Specifically, the bacterial community richness and diversity increased significantly, while the cross-species coexistence networks between the bacteria and fungi developed stronger modular structures and synergistic relationships. Furthermore, key functional groups (e.g., phosphoreleasing Actinobacteria and beneficial *Mortierella* fungi) were enriched, further driving elevated soil enzyme activity. This study provides systematic data for the first time on improving strongly acidic red soil using a continuous PT application and maize-potato rotation model, clarifying the interaction between soil properties, soil quality, and crop yields via microbial-mediated mechanisms. These findings offer crucial theoretical support for understanding the improvement effect associated with PTs since they show significant promise for effectively recycling solid waste and improving acidic soil. To ensure the long-term efficacy and environmental safety of this practice, the quality of the applied PTs should be continuously monitored, with particular attention to their trace element content. Future research can focus on the interaction between PTs and key functional microorganisms to establish a foundation for developing more efficient combined improvement technologies.

## Figures and Tables

**Table 1:** Basic soil properties before the experiment.

Soil Type	Organic Matter (g·kg^−1^)	AN (mg·kg^−1^)	AP (mg·kg^−1^)	AK (mg·kg^−1^)	pH	EA (cmol·kg^−1^)	ExK (cmol·kg^−1^)	ExNa (cmol·kg^−1^)	ExCa (cmol·kg^−1^)	ExMg (cmol·kg^−1^)
Upland Red Soil	26.67	145.25	24.44	309.50	4.67	3.31	1.12	0.72	3.06	0.28

## Data Availability

The data are contained within this article.

## Supplementary Materials

The following supporting information can be downloaded at: https://www.mdpi.com/article/10.3390/plants14223475/s1, Figure S1: Changes in soil chemical properties following phosphate tailing (PT) application; Figure S2: Effects of phosphate tailing (PT) application on the composition of soil bacterial and fungal communities; Figure S3: Effects of phosphate tailing (PT) application on bacterial-fungal co-occurrence networks in the rhizosphere soil of summer maize; Figure S4: Effects of phosphate tailing application on bacterial-fungal connections and key taxon composition; Figure S5: Correlations between bacterial/fungal communities and soil properties in the rhizosphere soil of summer maize after phosphate tailing (PT) application; Figure S6: Correlations between crop yield and soil quality index following phosphate tailings (PTs) application; Figure S7: Percentage increase in mean squared error (%IncMSE) of soil quality based on random forest models, showing the contribution rates of soil chemical, physical, and biological properties; Table S1: Effects of phosphate tailing (PT) application on bacterial and fungal communities and the topology of bacterial-fungal interkingdom networks in the rhizosphere soil of summer maize.

## References

[1] Teutscherova N., Vazquez E., Masaguer A., Navas M., Scow K.M., Schmidt R., Benito M. (2017). Comparison of Lime- and Biochar-Mediated PH Changes in Nitrification and Ammonia Oxidizers in Degraded Acid Soil. Biol. Fertil. Soils.

[2] Guo J.H., Liu X.J., Zhang Y., Shen J.L., Han W.X., Zhang W.F., Christie P., Goulding K.W.T., Vitousek P.M., Zhang F.S. (2010). Significant Acidification in Major Chinese Croplands. Science.

[3] Lu X., Mao Q., Gilliam F.S., Luo Y., Mo J. (2014). Nitrogen Deposition Contributes to Soil Acidification in Tropical Ecosystems. Glob. Chang. Biol..

[4] Schroder J.L., Zhang H., Girma K., Raun W.R., Penn C.J., Payton M.E. (2011). Soil Acidification from Long-Term Use of Nitrogen Fertilizers on Winter Wheat. Soil Sci. Soc. Am. J..

[5] Zhou J., Xia F., Liu X., He Y., Xu J., Brookes P.C. (2013). Effects of Nitrogen Fertilizer on the Acidification of Two Typical Acid Soils in South China. J. Soils Sediments.

[6] Bezdicek D. (2003). Subsoil Ridge Tillage and Lime Effects on Soil Microbial Activity, Soil PH, Erosion, and Wheat and Pea Yield in the Pacific Northwest, USA. Soil Tillage Res..

[7] Orton T.G., Mallawaarachchi T., Pringle M.J., Menzies N.W., Dalal R.C., Kopittke P.M., Searle R., Hochman Z., Dang Y.P. (2018). Quantifying the Economic Impact of Soil Constraints on Australian Agriculture: A Case-Study of Wheat. Land Degrad. Dev..

[8] Hijbeek R., van Loon M.P., Ouaret W., Boekelo B., van Ittersum M.K. (2021). Liming Agricultural Soils in Western Kenya: Can Long-Term Economic and Environmental Benefits Pay off Short Term Investments?. Agric. Syst..

[9] Uwiringiyimana E., Lai H., Ni N., Shi R., Pan X., Gao J., Biswash M.R., Li J., Cui X., Xu R. (2024). Comparative Efficacy of Alkaline Slag, Biomass Ash, and Biochar Application for the Amelioration of Different Acidic Soils. Plant Soil.

[10] Lollato R.P., Edwards J.T., Zhang H. (2013). Effect of Alternative Soil Acidity Amelioration Strategies on Soil PH Distribution and Wheat Agronomic Response. Soil Sci. Soc. Am. J..

[11] Bolan N., Sarmah A.K., Bordoloi S., Bolan S., Padhye L.P., Van Zwieten L., Sooriyakumar P., Khan B.A., Ahmad M., Solaiman Z.M. (2023). Soil Acidification and the Liming Potential of Biochar. Environ. Pollut..

[12] Yan P., Shen C., Zou Z., Fu J., Li X., Zhang L., Zhang L., Han W., Fan L. (2021). Biochar Stimulates Tea Growth by Improving Nutrients in Acidic Soil. Sci. Hortic..

[13] Pan X., Xu R., Nkoh J.N., Lu H., Hua H., Guan P. (2020). Effects of Straw Decayed Products of Four Crops on the Amelioration of Soil Acidity and Maize Growth in Two Acidic Ultisols. Environ. Sci. Pollut. Res..

[14] Liang F., Li B., Vogt R.D., Mulder J., Song H., Chen J., Guo J. (2023). Straw Return Exacerbates Soil Acidification in Major Chinese Croplands. Resour. Conserv. Recycl..

[15] Alsafasfeh A., Khodakarami M., Alagha L., Moats M., Molatlhegi O. (2018). Selective Depression of Silicates in Phosphate Flotation Using Polyacrylamide-Grafted Nanoparticles. Miner. Eng..

[16] Heuer S., Gaxiola R., Schilling R., Herrera-Estrella L., López-Arredondo D., Wissuwa M., Delhaize E., Rouached H. (2017). Improving Phosphorus Use Efficiency: A Complex Trait with Emerging Opportunities. Plant J..

[17] Wang B., Zhou Z., Xu D., Wu J., Yang X., Zhang Z., Yan Z. (2022). A New Enrichment Method of Medium–Low Grade Phosphate Ore with High Silicon Content. Miner. Eng..

[18] Yuan J.-H., E S.-Z., Che Z.-X. (2020). The Ameliorative Effects of Low-Grade Palygorskite on Acidic Soil. Soil Res..

[19] Tozsin G., Arol A.I., Cayci G. (2014). Evaluation of Pyritic Tailings from a Copper Concentration Plant for Calcareous Sodic Soil Reclamation. Physicochem. Probl. Miner. Process..

[20] Barker A.V. (2012). Plant Growth in Response to Phosphorus Fertilizers in Acidic Soil Amended with Limestone or Organic Matter. Commun. Soil Sci. Plant Anal..

[21] Venäläinen S.H., Nousiainen A., Kanerva S. (2024). The Potential of Phosphate Mine Tailings in the Remediation of Acidic Pb-Contaminated Soil. Soil Environ. Health.

[22] Venäläinen S.H., Nousiainen A., Silvennoinen M., Kanerva S. (2024). Stabilization of as and Sb in Contaminated Acidic Shooting Range Soil with Apatite Mine Tailings: Challenge of Co-Contamination. Soil Environ. Health.

[23] Li X., Chen D., Carrión V.J., Revillini D., Yin S., Dong Y., Zhang T., Wang X., Delgado-Baquerizo M. (2023). Acidification Suppresses the Natural Capacity of Soil Microbiome to Fight Pathogenic Fusarium Infections. Nat. Commun..

[24] Yadav S., Naresh R.K., Kumar Y., Yadav R.B. (2020). Conservation Tillage and Fertilization Impact on Carbon Sequestration and Mineralization in Soil Aggregates in the North West IGP under an Irrigated Rice-Wheat Rotation: A Review. Int. J. Curr. Microbiol. Appl. Sci..

[25] Peng J., Yang Q., Zhang C., Ni S., Wang J., Cai C. (2023). Aggregate Pore Structure, Stability Characteristics, and Biochemical Properties Induced by Different Cultivation Durations in the Mollisol Region of Northeast China. Soil Tillage Res..

[26] Dou X., Zhang C., Zhang J., Ma D., Chen L., Zhou G., Duan Y., Tao L., Chen J. (2024). Relationship Between Calcium Forms and Organic Carbon Content in Aggregates of Calcareous Soils in Northern China. Soil Tillage Res..

[27] Fageria N.K., Baligar V.C. Chapter 7 Ameliorating Soil Acidity of Tropical Oxisols by Liming for Sustainable Crop Production. https://www.sciencedirect.com/science/article/abs/pii/S0065211308004070.

[28] Zhang P., Chen X., Wei T., Yang Z., Jia Z., Yang B., Han Q., Ren X. (2016). Effects of Straw Incorporation on the Soil Nutrient Contents, Enzyme Activities, and Crop Yield in a Semiarid Region of China. Soil Tillage Res..

[29] Holland J.E., Bennett A.E., Newton A.C., White P.J., McKenzie B.M., George T.S., Pakeman R.J., Bailey J.S., Fornara D.A., Hayes R.C. (2018). Liming Impacts on Soils, Crops and Biodiversity in the UK: A Review. Sci. Total Environ..

[30] Han T., Cai A., Liu K., Huang J., Wang B., Li D., Qaswar M., Feng G., Zhang H. (2018). The Links Between Potassium Availability and Soil Exchangeable Calcium, Magnesium, and Aluminum Are Mediated by Lime in Acidic Soil. J. Soils Sediments.

[31] Jackson M.L., Luo J.N. (1986). Potassium-Release Mechanism on Drying Soils. Soil Sci..

[32] Tian J., Ge F., Zhang D., Deng S., Liu X. (2021). Roles of Phosphate Solubilizing Microorganisms from Managing Soil Phosphorus Deficiency to Mediating Biogeochemical P Cycle. Biology.

[33] Qin J., Jiang X., Yan Z., Zhao H., Zhao P., Yao Y., Chen X. (2023). Heavy Metal Content and Microbial Characteristics of Soil Plant System in Dabaoshan Mining Area, Guangdong Province. PLoS ONE.

[34] Ramut R., Jama-Rodzeńska A., Woźniak M., Siebielec S., Kamińska J., Szuba-Trznadel A., Gałka B. (2025). The Effect of Broadcast Struvite Fertilization on Element Soil Content and Microbial Activity Changes in Winter Wheat Cultivation in Southwest Poland. Sci. Rep..

[35] Bi R., Fu W., Fu X. (2024). Phosphorus Dynamics in Volcanic Soils of Weizhou Island, China: Implications for Environmental and Agricultural Applications. Environ. Geochem. Health.

[36] Ai C., Liang G., Sun J., He P., Tang S., Yang S., Zhou W., Wang X. (2015). The Alleviation of Acid Soil Stress in Rice by Inorganic or Organic Ameliorants Is Associated with Changes in Soil Enzyme Activity and Microbial Community Composition. Biol. Fertil. Soils.

[37] Wang T., Cao X., Chen M., Lou Y., Wang H., Yang Q., Pan H., Zhuge Y. (2022). Effects of Soil Acidification on Bacterial and Fungal Communities in the Jiaodong Peninsula, Northern China. Agronomy.

[38] Li F., Zhang S., Wang Y., Li Y., Li P., Chen L.X., Jie X., Hu D., Feng B., Yue K. (2020). Rare Fungus, Mortierella Capitata, Promotes Crop Growth by Stimulating Primary Metabolisms Related Genes and Reshaping Rhizosphere Bacterial Community. Soil Biol. Biochem..

[39] Shi A., Liu J., Zou S., Rensing C., Zhao Y., Zhang L., Xing S., Yang W. (2024). Enhancement of Cadmium Uptake in Sedum Alfredii Through Interactions Between Salicylic Acid/Jasmonic Acid and Rhizosphere Microbial Communities. Sci. Total Environ..

[40] Shi Y., Delgado-Baquerizo M., Li Y., Yang Y., Zhu Y., Peñuelas J., Chu H. (2020). Abundance of Kinless Hubs Within Soil Microbial Networks Are Associated with High Functional Potential in Agricultural Ecosystems. Environ. Int..

[41] Zhang C., Lei S., Wu H., Liao L., Wang X., Zhang L., Liu G., Wang G., Fang L., Song Z. (2024). Simplified Microbial Network Reduced Microbial Structure Stability and Soil Functionality in Alpine Grassland along a Natural Aridity Gradient. Soil Biol. Biochem..

[42] Luo Y., Ma L., Feng Q., Luo H., Chen C., Wang S., Yuan Y., Liu C., Cao X., Li N. (2024). Influence and Role of Fungi, Bacteria, and Mixed Microbial Populations on Phosphorus Acquisition in Plants. Agriculture.

[43] Cao Y., Shen Z., Zhang N., Deng X., Thomashow L.S., Lidbury I., Liu H., Li R., Shen Q., Kowalchuk G.A. (2024). Phosphorus Availability Influences Disease-Suppressive Soil Microbiome Through Plant-Microbe Interactions. Microbiome.

[44] Castro G.S.A., Crusciol C.A.C. (2013). Effects of Superficial Liming and Silicate Application on Soil Fertility and Crop Yield under Rotation. Geoderma.

[45] Tshiabukole J.P.K., Khonde G.P., Phongo A.M., Ngoma N., Vumilia R.K., Kankolongo A.M. (2022). Liming and Mineral Fertilization of Acid Soils in Maize Crop Within the Savannah of Southwestern of Democratic Republic of Congo. OALib.

[46] Zhao W., Cai Z., Xu Z. (2007). Does Ammonium-Based N Addition Influence Nitrification and Acidification in Humid Subtropical Soils of China?. Plant Soil.

[47] Ur Rahman S., Han J.-C., Ahmad M., Ashraf M.N., Khaliq M.A., Yousaf M., Wang Y., Yasin G., Nawaz M.F., Khan K.A. (2024). Aluminum Phytotoxicity in Acidic Environments: A Comprehensive Review of Plant Tolerance and Adaptation Strategies. Ecotoxicol. Environ. Saf..

[48] Zhou X., Tahvanainen T., Malard L., Chen L., Pérez-Pérez J., Berninger F. (2024). Global Analysis of Soil Bacterial Genera and Diversity in Response to PH. Soil Biol. Biochem..

[49] Shi W., He Z., Lu J., Wang L., Guo J., Qiu S., Ge S. (2024). Response of Nitrifiers to Gradually Increasing PH Conditions in a Membrane Nitrification Bioreactor: Microbial Dynamics and Alkali-Resistant Mechanism. Water Res..

[50] Bach E.M., Hofmockel K.S. (2014). Soil Aggregate Isolation Method Affects Measures of Intra-Aggregate Extracellular Enzyme Activity. Soil Biol. Biochem..

[51] Ju W., Fang L., Shen G., Delgado-Baquerizo M., Chen J., Zhou G., Ma D., Bing H., Liu L., Liu J. (2023). New Perspectives on Microbiome and Nutrient Sequestration in Soil Aggregates during Long-Term Grazing Exclusion. Glob. Change Biol..

[52] Bao S. (2010). Analysis of Agrochemical Soil.

[53] Xu D., Ma W., Chen S., Jiang Q., He K., Shi Z. (2018). Assessment of Important Soil Properties Related to Chinese Soil Taxonomy Based on Vis–NIR Reflectance Spectroscopy. Comput. Electron. Agric..

[54] Tiruneh G.A., Hanjagi A., Phaneendra B., Lalitha M., Vasundhara R., Ramamurty V., Abdul Rahaman S., Ravikiran T., Simegn A.A., Addis T.M. (2024). Pedogenic Variables with Color Indices of Rubified Alfisols in the Kakalachinte Microwatershed, Karnataka, South India. Geoderma Reg..

[55] Zhang G.-L., Gong Z.-T. (2012). Soil Survery Laboratory Methods.

[56] Chen L., Jiang Y., Liang C., Luo Y., Xu Q., Han C., Zhao Q., Sun B. (2019). Correction to: Competitive Interaction with Keystone Taxa Induced Negative Priming under Biochar Amendments. Microbiome.

[57] Chen C., Wang M., Zhu J., Tang Y., Zhang H., Zhao Q., Jing M., Chen Y., Xu X., Jiang J. (2022). Long-Term Effect of Epigenetic Modification in Plant–Microbe Interactions: Modification of DNA Methylation Induced by Plant Growth-Promoting Bacteria Mediates Promotion Process. Microbiome.

[58] Deng Y., Jiang Y.-H., Yang Y., He Z., Luo F., Zhou J. (2012). Molecular Ecological Network Analyses. BMC Bioinform..

[59] Jiang Y., Sun B., Li H., Liu M., Chen L., Zhou S. (2015). Aggregate-Related Changes in Network Patterns of Nematodes and Ammonia Oxidizers in an Acidic Soil. Soil Biol. Biochem..

[60] Li N., Wen S., Wei S., Li H., Feng Y., Ren G., Yang G., Han X., Wang X., Ren C. (2021). Straw Incorporation plus Biochar Addition Improved the Soil Quality Index Focused on Enhancing Crop Yield and Alleviating Global Warming Potential. Environ. Technol. Innov..

[61] Vanalle R.M., Ganga G.M.D., Godinho Filho M., Lucato W.C. (2017). Green Supply Chain Management: An Investigation of Pressures, Practices, and Performance Within the Brazilian Automotive Supply Chain. J. Clean. Prod..

