# Supplementary material for: Research on Utilizing Phosphorus Tailing Recycling to Improve Acidic Soil: The Synergistic Effect on Crop Yield, Soil Quality, and Microbial Communities
Source: Plants (Basel). 2025 Nov 14;14(22):3475. doi: 10.3390/plants14223475 (PMC12656315; doi:10.3390/plants14223475)
Supplement: Supplementary file 1 [file plants-14-03475-s001.zip › plants-3903858-supplementary.pdf]

Supplementary material to

# Research on Utilizing Phosphorus Tailing Recycling to Improve Acidic Soil: The Synergistic Effect on Crop Yield, Soil Quality, and Microbial Communities

**Chuanxiong Geng [1,†], Huineng Shi [2,†], Jinghui Wang [2], Huimin Zhang [2], Xinling Ma [1,2], Jinghua Yang [1], Xi Sun [1], Yupin Li [2], Yi Zheng [2,3,*] and Wei Fan [2,*]**

[1] Institute of Agricultural Environment and Resources, Yunnan Academy of Agricultural Sciences,
Kunming 650201, China; gengchuanxiong@163.com (C.G.); 15559898127@163.com (X.M.); yjh699@126.com (J.Y.); sunxi_36@163.com (X.S.)

[2] College of Resources and Environment, Yunnan Agricultural University, Kunming, 650201, China;
shihuineng97@163.com (H.S.); 18213704161@163.com (J.W.); zhanghuimin115zhm@126.com (H.Z.);
liyupin2021@163.com (Y.L.)

[3] Yunnan Open University, Kunming 650500, China

* Correspondence: zhengyi-64@163.com (Y.Z.); fanwei1128@aliyun.com (W.F.)

† These authors contributed equally to this work.

## Supplementary table

Table S1. Effects of phosphate tailing (PT) application on bacterial and fungal communities and the topology of bacterial-fungal interkingdom networks in the rhizosphere soil of summer maize.

| Co-network types | Topology properties | CK | PTs-1 | PTs-2 | PTs-3 |
|---|---|---|---|---|---|
| Bacteria | Average degree | 23.31 | 20.64 | 31.32 | 21.57 |
| | Average path length | 3.49 | 3.55 | 3.33 | 3.44 |
| | Average cluster coefficient | 0.59 | 0.57 | 0.60 | 0.54 |
| | Modularity | 0.60 | 0.61 | 0.49 | 0.59 |
| Fungi | Average degree | 5.70 | 4.50 | 3.25 | 6.27 |
| | Average path length | 4.31 | 4.80 | 4.34 | 3.90 |
| | Average cluster coefficient | 0.55 | 0.72 | 0.62 | 0.56 |
| | Modularity | 0.67 | 0.76 | 0.71 | 0.62 |
| Interkingdom | Average degree | 28.29 | 24.52 | 33.19 | 27.39 |
| | Average path length | 3.46 | 3.51 | 3.24 | 3.42 |
| | Average cluster coefficient | 0.57 | 0.57 | 0.58 | 0.55 |
| | Modularity | 0.57 | 0.58 | 0.52 | 0.56 |

# Supplementary figure

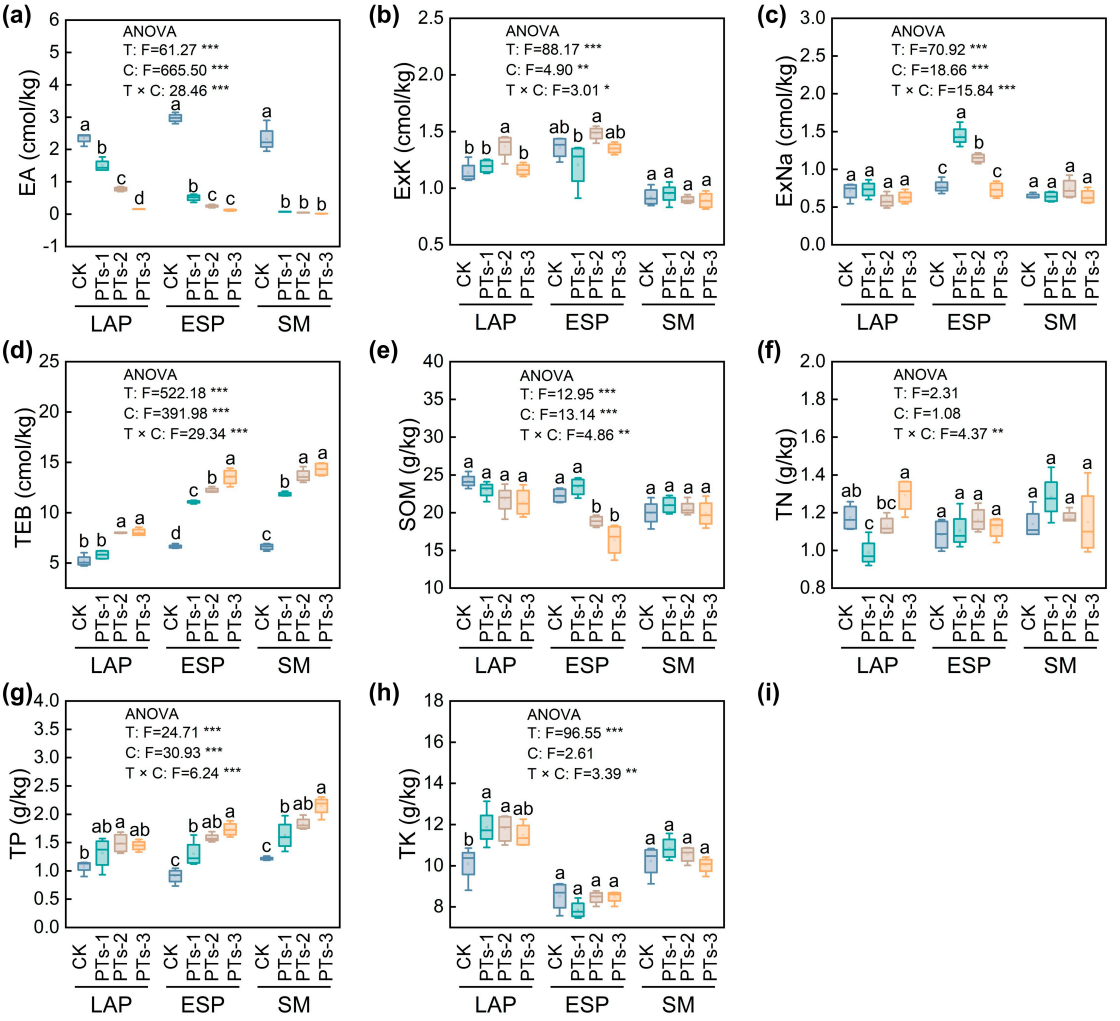


**Figure S1.** Changes in soil chemical properties following phosphate tailing (PT) application. LAP, late autumn potato; ESP, early spring potato; SM, summer maize. T, different treatments; C, cropping system. EA: exchangeable acidity; ExK: exchangeable potassium; ExNa: exchangeable sodium; TEB: total exchangeable bases; SOM: soil organic matter; TN: total nitrogen; TP: total phosphorus; TK: total potassium. Error bars represent standard error of the mean (n = 4). Different lowercase letters indicate significant differences between treatments ($p < 0.05$). *, $p < 0.05$, **, $p < 0.01$ and *, $p < 0.001$.

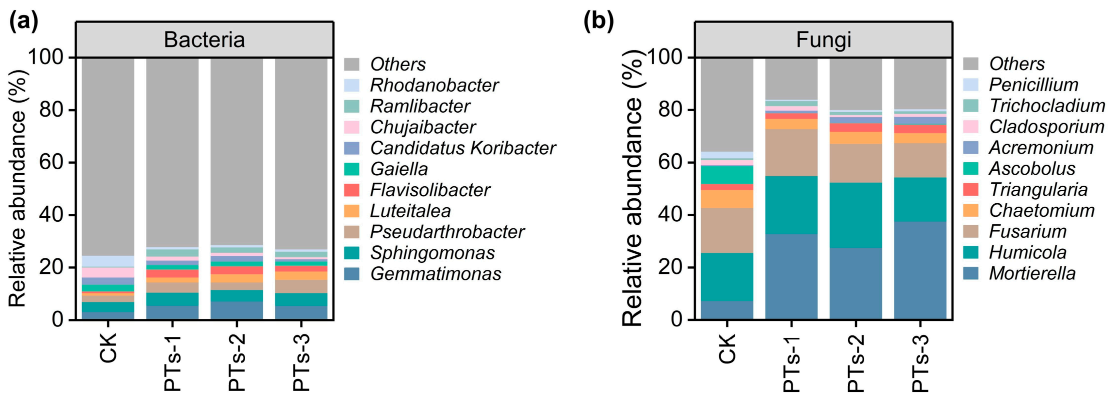


**Figure S2.** Effects of phosphate tailing (PT) application on the composition of soil bacterial and fungal communities. (a) Bacterial composition at the genus level; (b) Fungal composition at the genus level.

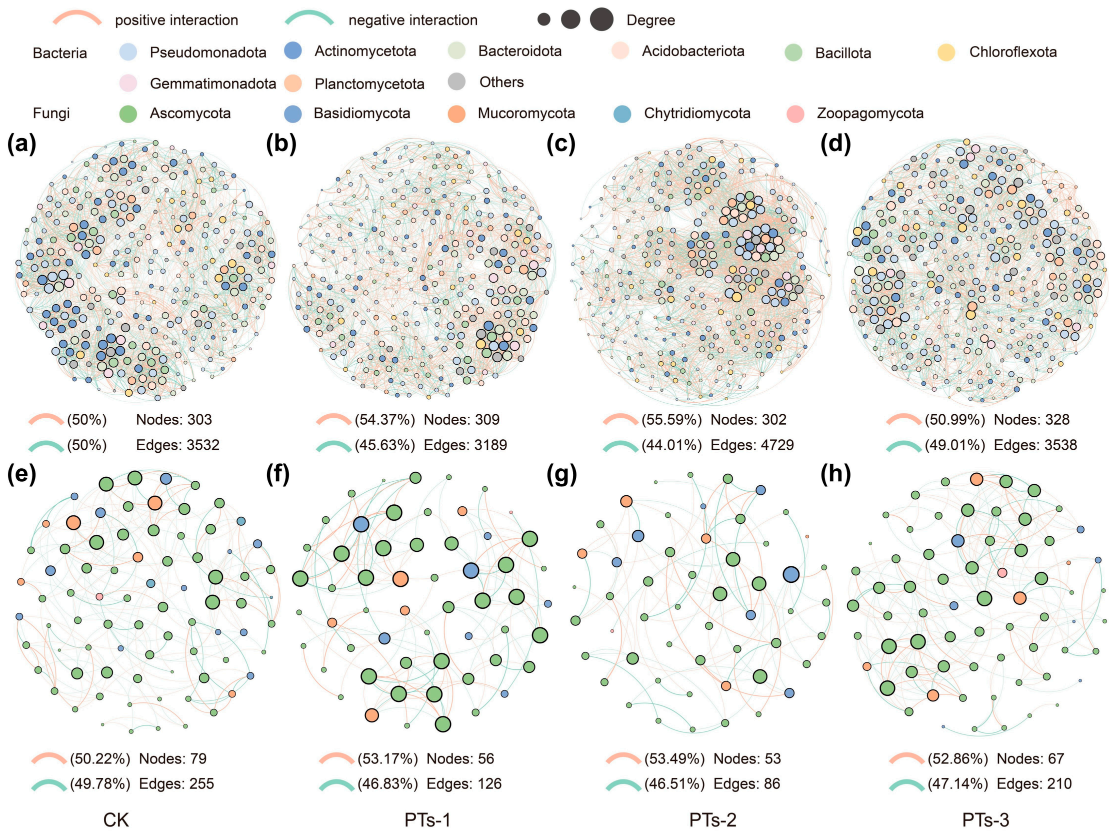


**Figure S3.** Effects of phosphate tailing (PT) application on bacterial-fungal co-occurrence networks in the rhizosphere soil of summer maize. Bacterial co-occurrence networks (a–d); fungal co-occurrence networks (e–h). Edges in light yellow and light green represent positive and negative connections, respectively. Networks are colored by bacterial phyla and fungal classes, with nodes representing different ASVs (amplicon sequence variants) of bacteria or fungi. Node size is proportional to the number of connections (i.e., degree).

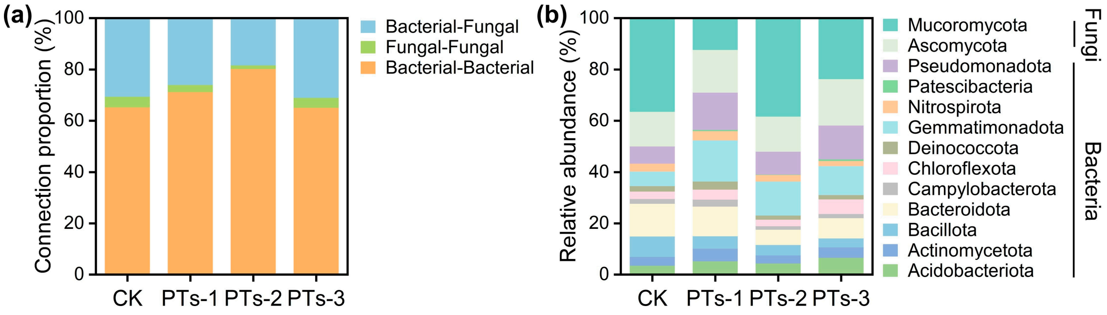


**Figure S4.** Effects of phosphate tailing application on bacterial-fungal connections and key taxon composition. (a) Relationships between bacteria-bacteria, fungi-fungi, and bacteria-fungi interactions; (b) Composition of key taxa.

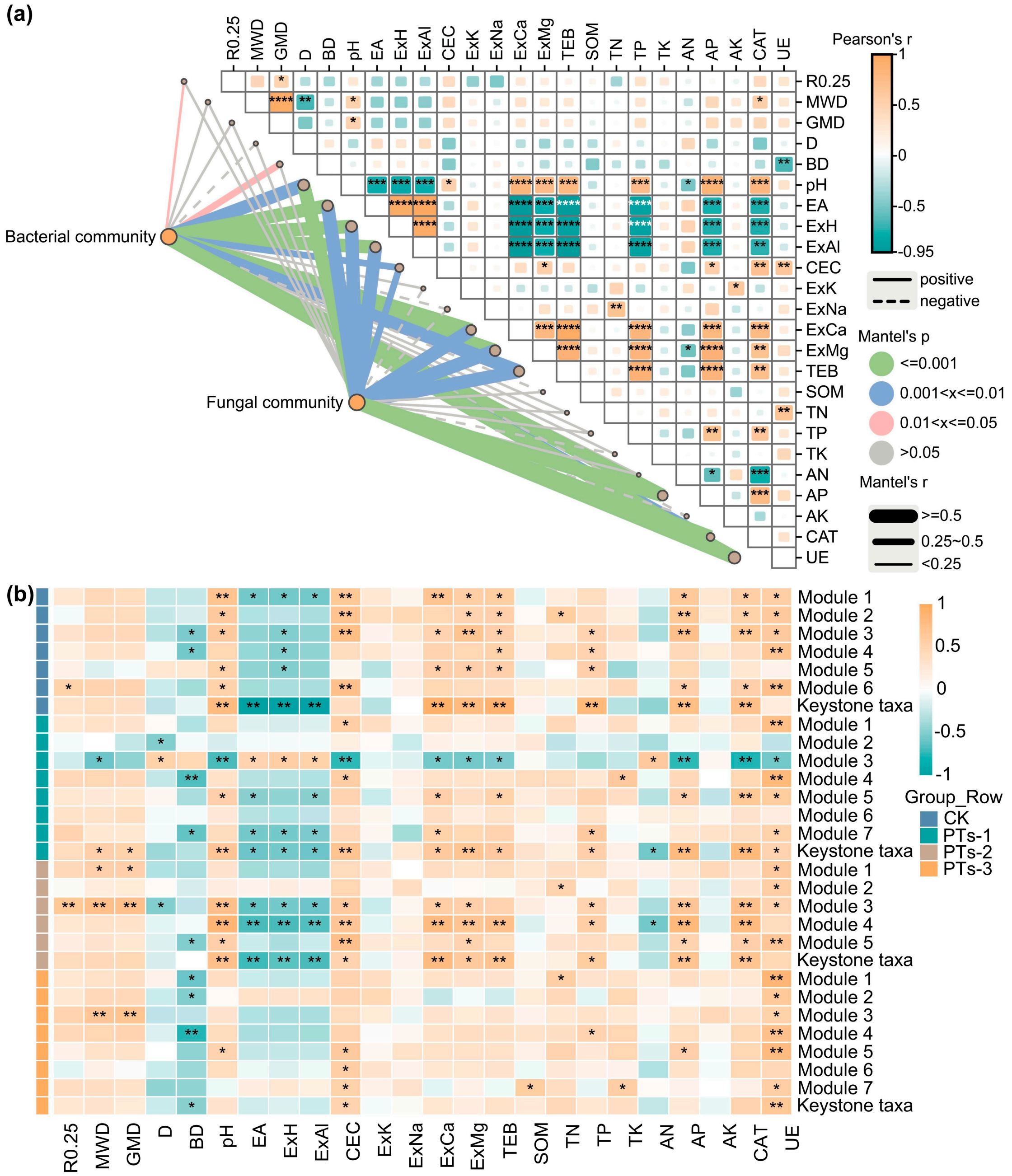


**Figure S5.** Correlations between bacterial/fungal communities and soil properties in the rhizosphere soil of summer maize after phosphate tailing (PT) application. (a) Relationships between bacterial and fungal community composition and environmental factors based on Mantel test analysis. (b) Spearman's correlation analysis between the relative abundance of modules and key taxa in the bacterial-fungal interkingdom co-occurrence network and environmental factors. Bacterial and fungal compositions are represented by the first principal coordinate (PCo1). R0.25: mass percentage of soil aggregates with particle size >0.25 mm; MWD: mean weight

diameter; GMD: geometric mean diameter; D: fractal dimension; BD: bulk density; pH: soil pH; EA: exchangeable acidity; ExH: exchangeable hydrogen; ExAl: exchangeable aluminum; CEC: cation exchange capacity; ExK: exchangeable potassium; ExNa: exchangeable sodium; ExCa: exchangeable calcium; ExMg: exchangeable magnesium; TEB: total exchangeable bases; SOM: soil organic matter; TN: total nitrogen; TP: total phosphorus; TK: total potassium; AN: available nitrogen; AP: available phosphorus; AK: available potassium; CAT: soil catalase; UE: soil urease. Soil quality and crop yield were correlated with various factors based on Mantel tests using Bray-Curtis distance. Edge width corresponds to Mantel's r statistic, and edge color indicates statistical significance based on 999 permutation tests. The color gradient represents Spearman's correlation coefficient for pairwise correlations between factors. *, $p < 0.05$, **, $p < 0.01$ and ***, $p < 0.001$.

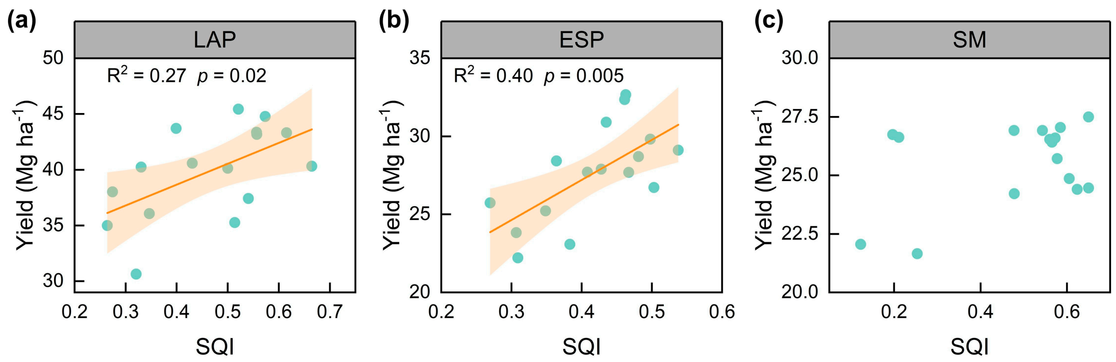


**Figure S6.** Correlations between crop yield and soil quality index following phosphate tailings (PTs) application. (a) Relationship between late autumn potato yield and soil quality; (b) Relationship between early spring potato yield and soil quality; (c) Relationship between summer maize yield and soil quality. LAP, late autumn potato; ESP, early spring potato; SM, summer maize.

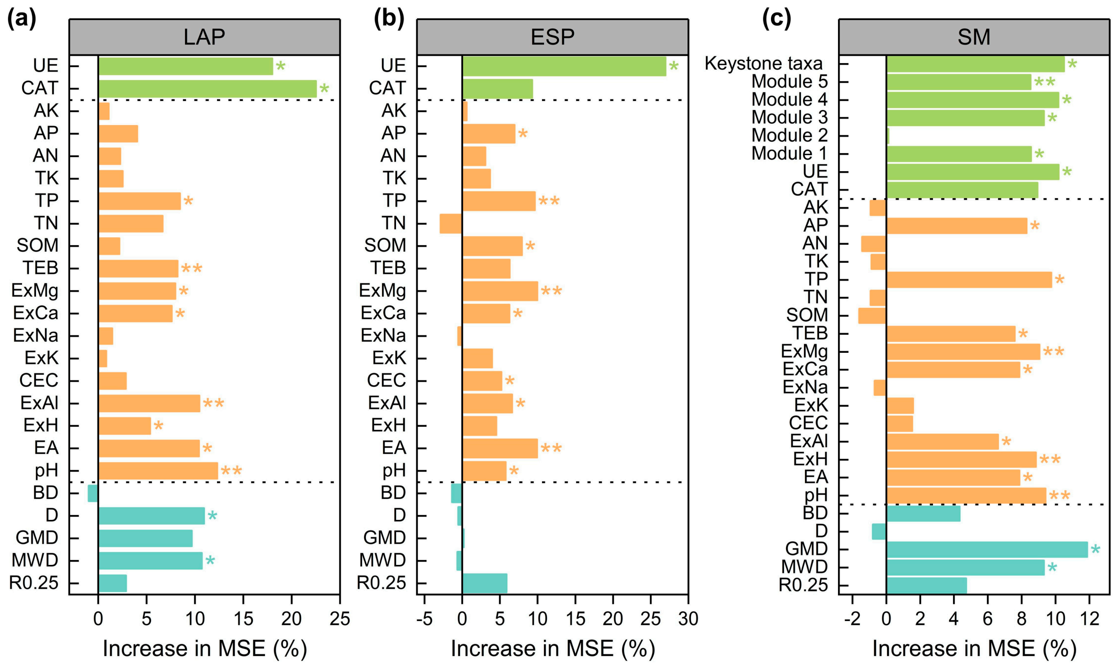


**Figure S7.** Percentage increase in mean squared error (%IncMSE) of soil quality based on random forest models, showing the contribution rates of soil chemical, physical, and biological properties. LAP, late autumn potato; ESP, early spring potato; SM, summer maize. R0.25: mass percentage of soil aggregates >0.25 mm; MWD: mean weight diameter; GMD: geometric mean diameter; D: fractal dimension; BD: bulk density; pH: soil pH; EA: exchangeable acidity; ExH: exchangeable hydrogen; ExAl: exchangeable aluminum; CEC: cation exchange capacity; ExK: exchangeable potassium; ExNa: exchangeable sodium; ExCa: exchangeable calcium; ExMg: exchangeable magnesium; TEB: total exchangeable bases; SOM: soil organic matter; TN: total nitrogen; TP: total phosphorus; TK: total potassium; AN: available nitrogen; AP: available phosphorus; AK: available potassium; SCAT: soil catalase activity; SUE: soil urease activity. Key taxa and network modules are represented by the first principal coordinate (PCo1) values. *, $p < 0.05$, **, $p < 0.01$ and ***, $p < 0.001$.
